# BMI, All-Cause and Cause-Specific Mortality in Chinese Singaporean Men and Women: The Singapore Chinese Health Study

**DOI:** 10.1371/journal.pone.0014000

**Published:** 2010-11-15

**Authors:** Andrew O. Odegaard, Mark A. Pereira, Woon-Puay Koh, Myron D. Gross, Sue Duval, Mimi C. Yu, Jian-Min Yuan

**Affiliations:** 1 Division of Epidemiology and Community Health, School of Public Health, University of Minnesota, Minneapolis, Minnesota, United States of America; 2 Department of Epidemiology and Public Health, Yong Loo Lin School of Medicine, National University of Singapore, Singapore, Singapore; Indiana University, United States of America

## Abstract

**Background:**

The optimal range of relative weight for morbidity and mortality in Asian populations is an important question in need of more thorough investigation, especially as obesity rates increase. We aimed to examine the association between body mass index (BMI), all cause and cause-specific mortality to determine the optimal range of BMI in relation to mortality in Chinese men and women in Singapore.

**Methodology/Principal Findings:**

We analyzed data from a prospective cohort study of 51,251 middle-aged or older (45–74) Chinese men and women in the Singapore Chinese Health Study. Participants were enrolled and data on body weight and covariates were collected in 1993–1998 and participants were followed through 2008. The analysis accounted for potential methodological issues through stratification on smoking and age, thorough adjustment of demographic and lifestyle confounders and exclusion of deaths early in the follow-up.

**Conclusions/Significance:**

Increased risk of mortality was apparent in underweight (<18.5) and obese BMI categories (≥27.5) independent of age and smoking. Regardless of age or BMI, smoking considerably increased the rate of mortality and modified the association between BMI and mortality. The most favorable range of BMI for mortality rates and risk in non-smoking persons below age 65 was 18.5–21.4 kg/m^2^, and for non-smoking persons aged 65 and above was 21.5–24.4 kg/m^2^.

## Introduction

The prevalence of obesity continues to increase in developed and developing Asian nations, and the rates of chronic disease have mirrored this trend. [1] With increasing global obesity trends, heightened attention has been paid to the relation between body weight and mortality. Many studies on this topic, mostly on Western populations, have produced somewhat divergent results. [2] However, a recent analysis of over 900,000 adults primarily from prospective cohorts of Western populations found that the most favorable Body Mass Index (BMI) range in relation to the lowest all-cause mortality rate is 22.5 to 25.0 kg/m^2^. [3] By contrast, inconsistent associations have been observed in the few prospective Asian studies that have been reported. [4]–[9]

There are multiple methodological considerations in the study of body weight and mortality. The great majority of studies have used BMI (kg/m^2^), which is a reasonable and easily applicable surrogate measure of adiposity in populations, [10], [11] but subject to criticism. [12] The main methodological issues beyond the measure of adiposity focus on accounting for the anorectic effects of cigarette smoking, [13], [14] confounding due to prevalent and antecedent disease, [14]–[16] control of intermediate variables in the causal pathway, [10], [14] and how age may modify the association. [14] For example, BMI is a less reliable marker of adiposity due to differential loss of muscle and bone mass in persons aged 65 years and above. [14], [17]

The investigation of BMI and mortality in Asians presents an intriguing study due to the larger proportion of the population with low BMI's, and research on Asian populations is comparatively sparse. Furthermore, South/Southeast Asians appear to be distinctive anthropometrically compared with Western populations; with lower BMI's, but higher body fat percentages comparable to World Health Organization (WHO) BMI strata for overweight or obesity in Western populations, and greater abdominal and visceral fat deposition. [18]–[20] Thus, the discussion of an optimal weight range or the most favorable BMI-range in relation to the lowest mortality rate and risk in Asian populations is distinct from the discussion on Western populations.

The Singapore Chinese Health Study (SCHS) is a prospective cohort investigation of diet and cancer in 63,257 Chinese men and women in Singapore. The study affords the ability to account for potential methodological issues through stratification on smoking and age, thorough adjustment of demographic and lifestyle confounders, and exclusion of prevalent disease and early deaths. Therefore, the aim of this study was to examine the association between BMI and all-cause and cause specific mortality using the aforementioned methodological approaches to determine the optimal range of BMI in a large sample of Chinese men and women living in Singapore.

## Methods

### Study Population

The design of the Singapore Chinese Health Study has been previously described. [20] Briefly, the cohort was drawn from men and women, aged 45 to 74 at enrollment, who belonged to one of the major dialect groups (Hokkien or Cantonese) of Chinese in Singapore. Between April 1993 and December 1998, 63,257 individuals completed an in-person interview that included questions on demographics, educational attainment, height, weight, use of tobacco and alcohol, usual physical activity, menstrual and reproductive history (women only), medical history, family history of cancer and a 165-item food frequency section assessing usual dietary intake of the previous year. The institutional review boards at the National University of Singapore and the University of Minnesota approved this study. Written informed consent was obtained from all participants.

### Exposure Assessment

Self reported height and weight were collected at the baseline interview. BMI was calculated as weight (kg) divided by height squared (m). Self-report of body weight and height has been shown to be highly valid across many populations [10], as well as specifically in Asians. [21] Age was defined as age in years at the time of the baseline examination. Education was categorized into no formal education, primary school, and secondary school or above. Cigarette smoking was classified into never smoker, former smoker, and current smoker as described previously. [22] Participants reporting a history of physician diagnosed cardiovascular disease, diabetes, or respiratory disease at baseline were classified as having a history of prevalent disease for the present analysis. Physical activity was assessed as the amount of time spent doing strenuous sports (e.g. jogging) and moderate activity (e.g. brisk walking) in a week, but due to low activity levels in the population, participants reporting any strenuous sporting activity or moderate activity were combined for the present analysis.

A semi-quantitative food frequency questionnaire specifically developed for this population assessing 165 commonly consumed food items was administered during the baseline interview. The questionnaire has subsequently been validated against a series of 24-hour dietary recall interviews, [20] as well as selected biomarkers. [23], [24] Dietary patterns were derived for this study population using principal component analysis including all 165 foods and beverages as described previously. [25] Briefly, the aim of PCA in nutritional analyses is to account for the maximal variance of dietary intake by combining the many different dietary variables into a smaller number of factors based upon the intercorrelations of these variables. A vegetable, fruit, and soy rich pattern characterized by high intake of those respective foods and lower intake of meats, dim sum, western fast food and soft drinks was included as a covariate. Frequency of alcohol intake as the summation of beer, rice wine, other wine and hard liquor was considered as an individual variable and grouped as nondrinker/monthly drinker, weekly drinker, and daily drinker.

### Assessment of Mortality

Information on date and cause of death was obtained through linkage analysis with the nation-wide registry of birth and death in Singapore. Up to six different international classification of disease codes version 9 (ICD-9) were recorded in the registry. Primary cause of death was used for analysis. Vital status for cohort participants was updated through December 31, 2008. As of April 2008 only 27 persons were lost to follow up due to migration out of Singapore, suggesting that emigration of the cohort participants was negligible and that vital statistics follow up was virtually complete.

The end points in our cause-specific analyses were deaths from cardiovascular disease (codes 394.0–459.0), ischemic heart disease (410.0–414.9, 427.5), and cerebrovascular disease (430.0–438.0), all cancers (140.0–195.8 and 199–208.9), and excess weight related cancers. Specific cancer sites related to excess weight included esophagus (150.0–150.9), stomach (151.0–151.9), colon (153.0–153.9), liver (155.0–155.2), gallbladder (156.0–156.9), pancreas (157.0–157.9), female breast (174.0–174.9), cervix and corpus uteri (179, 182.0–182.8), ovary (183.0–183.9), prostate (185), and kidney (189.0–189.9).

### Statistical Analysis

Of the original 63,257 participants, we excluded 1,936 subjects with a history of invasive cancer (except non-melanoma skin cancer) or superficial, papillary bladder cancer at baseline since they did not meet study inclusion criteria, and 10,070 participants missing either or both height and weight measures. The present analyses included 51,251 participants. Participants excluded due to missing BMI (N = 10,070) were not materially different across the noted demographic and lifestyle characteristics according to smoking status compared to participants with full data and included in the analysis (51,251).

Study participants were grouped according to 8 categories of BMI, as reported at the baseline interview (<18.5, 18.5–19.9, 20.0–21.4, 21.5–22.9, 23.0–24.4, 24.5–25.9, 26.0–27.4, ≥27.5). These categories were created to allow for a detailed examination of the association between BMI and all-cause and cause-specific mortality based on the distribution of BMI in the study population with the consideration of BMI cut points recommended by the World Health Organization (WHO) working group for Asian populations (BMI<18.5 = underweight, 18.5–22.9 = normal weight, 23.0–27.4 = overweight, ≥27.5 = obese). [26] We further separated individuals with BMI's<18.5 into the BMI groups of 17.0–18.4 and <17.0 to examine the low end of the BMI spectrum. For each study subject, person-years were counted from the date of baseline interview to the date of death, date of last contact (for the few subjects who migrated out of Singapore) or December 31, 2008, whichever occurred first. Baseline characteristics were calculated for participants across each category of BMI. Age and sex standardized mortality rates were calculated using the person-year weight of the entire cohort during the follow-up by the following age categories: (<50, 50–54, 55–59, 60–64, 65–69, 70+).

Proportional hazards (Cox) regression methods were used to examine the associations between BMI and hazard risk of death. All regression analyses were conducted using SAS statistical software version 9.1 (SAS institute, Cary, NC). We estimated the hazard ratio (HR) of death for levels of BMI and the corresponding 95% confidence interval (CI). There was no evidence that proportional hazards assumptions were violated as indicated by the lack of significant interaction between BMI and a function of survival time in the models. The referent BMI category was chosen based on the lowest age and sex standardized mortality rate. Our primary Cox regression model included the following covariates: Age and its quadratic term (age^2^), sex, year of interview (1993–95 and 1996–98), dialect (Hokkien vs. Cantonese), level of education (no formal schooling, primary school, secondary school or above), any moderate or strenuous physical activity (yes vs. no), and quintile of vegetable, fruit, and soy rich dietary pattern score. In smokers we further adjusted for number of cigarettes smoked per day and number of years of smoking over lifetime, plus duration of time quit in non-smokers. The presence of a quadratic BMI-mortality association (the U or J-shaped curve) was examined by including a quadratic term of the median BMI value within each BMI category in the Cox regression models. Separate product terms of smoking, prevalent disease, age and sex with BMI were included in Cox regression models to examine potential interactions. To reduce potential bias due to preexisting disease or illness-related weight loss, all analyses were repeated after excluding subjects with reported prevalent disease (CVD, diabetes, respiratory) at baseline and mortality incidence within the first 5 years post-enrollment. Alcohol intake as an independent variable did not predict risk of death alone or in combination with a dietary pattern score not encompassing alcohol intake, so it was not included in the final regression model for the present analysis. Further text and figures from a non-parametric (local polynomial) regression analysis can be found in **File S1**.

## Results

Baseline characteristics of the study participants according to eight categories of BMI by smoking status are presented in **Table 1**. There is evidence that the relation between BMI and mortality in smokers and non-smokers differs in important ways (P interaction between smoking and BMI = 0.0012). Hence, results are stratified by smoking status category. Population trends show males smoked at significantly higher levels than females in the study population, as 86% of ever-smokers were male. Greater education, physical activity and a higher vegetable, fruit, and soy rich dietary pattern score all had a significant inverse association with mortality.

**Table 1:** Baseline characteristics by smoking status according to body mass index (BMI) in SCHS.

	Body Mass Index (kg/m^2^)							
Characteristics	<18.5	18.5–19.9	20.0–21.4	21.5–22.9	23.0–24.4	24.5–25.9	26.0–27.4	≥27.5
**Non-smokers, N = 35,766**								
N	2,260	3,612	5,643	6,422	6,210	4,779	2,937	3,903
Age	55.5 (8.1)	54.8 (7.7)	54.8 (7.8)	54.9 (7.7)	55.1 (7.6)	55.4 (7.7)	55.3 (7.7)	55.6 (7.8)
Sex (% women)	75.7	76.0	72.1	71.5	68.9	70.2	67.3	74.6
Weight (kg)	44.0 (5.0)	48.5 (4.7)	52.4 (5.1)	55.9 (5.4)	59.7 (5.7)	63.1 (6.1)	66.9 (6.7)	73.3 (9.8)
Weight men (kg)	48.7 (4.6)	53.4 (4.3)	57.4 (4.5)	61.4 (4.6)	65.1 (5.0)	69.1 (5.2)	73.0 (5.8)	80.5 (10.1)
Weight women (kg)	42.5 (4.2)	47.0 (3.6)	50.5 (3.9)	53.6 (3.8)	57.3 (4.2)	60.6 (4.4)	63.9 (4.8)	70.9 (8.4)
Height (cm)	159.3 (8.2)	158.2 (7.3)	158.6 (7.4)	158.3 (7.3)	158.5 (7.4)	157.9 (7.5)	158.1 (7.8)	156.2 (7.9)
Height men (cm)	167.2 (7.2)	166.0 (6.5)	165.9 (6.2)	166.0 (6.0)	165.6 (6.1)	165.3 (6.1)	165.2 (6.3)	164.1 (6.8)
Height women (cm)	156.7 (6.7)	155.8 (5.7)	155.7 (5.7)	155.3 (5.3)	155.3 (5.4)	154.7 (5.5)	154.6 (5.8)	153.5 (6.2)
Body mass index	17.3 (1.0)	19.3 (0.4)	20.8 (0.4)	22.2 (0.4)	23.7 (0.4)	25.2 (0.5)	26.7 (0.4)	30.0 (2.8)
Education (% secondary)	34.8	37.3	36.6	34.2	33.4	29.1	29.7	24.9
∞Any physical activity (%)	26.3	29.8	30.1	31.8	31.1	31.3	30.6	25.2
Alcoholic drinks/wk	0.4 (1.9)	0.4 (2.2)	0.5 (2.5)	0.4 (2.2)	0.4 (2.1)	0.4 (2.1)	0.5 (2.6)	0.5 (3.0)
¥Hypertension (%)	10.2	12.9	16.4	21.4	26.6	31.3	35.5	42.2
§Prevalent disease (%)	10.4	9.4	10.9	11.5	13.7	13.9	15.0	18.1
								
**Ever-smokers, N = 15,485**								
N	1,590	1,860	2,590	2,585	2,513	1,834	1,100	1,413
Age	59.5 (7.9)	58.2 (8.1)	58.0 (7.9)	57.9 (8.1)	57.3 (7.8)	57.2 (7.9)	57.2 (7.8)	56.4 (7.8)
Sex (% women)	17.9	14.8	13.0	15.6	12.4	12.5	13.1	18.1
Weight (kg)	46.7 (5.2)	52.0 (4.8)	55.9 (4.9)	59.9 (5.4)	64.1 (5.7)	68.0 (5.9)	71.7 (6.2)	78.6 (9.8)
Weight men (kg)	47.9 (4.5)	52.9 (4.3)	56.9 (4.4)	61.1 (4.8)	65.1 (5.1)	69.1 (5.3)	72.8 (5.5)	80.2 (9.1)
Weight women (kg)	41.1 (4.6)	46.6 (3.8)	49.9 (3.8)	53.3 (3.7)	56.9 (4.4)	60.1 (4.2)	64.0 (4.9)	71.3 (9.5)
Height (cm)	164.5 (7.9)	164.0 (7.5)	163.9 (7.0)	163.8 (7.2)	164.2 (7.2)	163.9 (7.1)	163.7 (7.1)	162.3 (7.6)
Height men (cm)	166.4 (6.5)	165.5 (6.6)	165.3 (6.2)	165.5 (6.2)	165.5 (6.2)	165.3 (6.1)	165.0 (6.2)	164.3 (6.3)
Height women (cm)	155.7 (7.5)	155.2 (6.3)	155.0 (5.6)	154.8 (5.1)	154.7 (5.9)	154.1 (5.2)	154.7 (5.8)	153.2 (5.9)
Body mass index	17.2 (1.1)	19.3 (0.4)	20.8 (0.4)	22.3 (0.4)	23.7 (0.5)	25.3 (0.4)	26.7 (0.4)	29.8 (3.0)
Education (% secondary)	22.5	27.6	28.3	29.7	33.3	31.6	29.9	26.4
∞Any physical activity (%)	21.7	24.4	27.4	28.6	31.0	30.2	32.8	26.8
Alcoholic drinks/wk	2.8 (7.7)	2.6 (7.5)	2.5 (7.2)	2.1 (5.9)	2.1 (6.1)	1.9 (6.1)	2.1 (6.8)	1.9 (6.0)
¥Hypertension (%)	9.4	11.6	15.3	19.7	25.6	31.7	35.3	38.7
§Prevalent disease (%)	13.4	13.7	15.9	16.9	19.1	20.5	22.5	22.5

All values mean (standard deviation) or percentage of population (%).

§ Prevalent disease =  Self reported physician diagnosed baseline cardiovascular disease, diabetes mellitus or respiratory disease.

∞ Any physical activity (%)  =  Report of any moderate or strenuous leisure physical activity.

¥ Hypertension – Self reported physician diagnosed.

In 9,777 current smokers with a mean follow up of 11.8 years, there were 2,762 deaths. There is a statistically significant quadratic association between BMI and all-cause mortality in current smokers with the lowest age and sex standardized mortality rate among those with BMI's 26–27.4 as presented in **Table S1**. In 5,708 ex-smokers, during a mean 11.6 years of follow up there were 1,423 deaths. The lowest standardized mortality rate was among those with BMI's 23–24.4 and a significant quadratic association between BMI and risk of mortality is observed. In 35,766 non-smokers (4,590 with reported prevalent disease), during a mean follow up of 12.7 years, there were 4,171 deaths. The lowest age and sex standardized mortality rate is observed in BMI's 18.5–19.9 kg/m^2^ and risk increases at BMI's>26.0 and <18.5.

The remaining results presented on cause-specific and stratified analyses are focused on non-smokers who did not report a surveyed chronic disease or health condition at baseline nor died within five years. This analytic approach may best inform on the optimal relative weight range for public health purposes; [14] or the most favorable BMI-range in non-smokers in relation to the lowest mortality rate and risk.

**Figure 1:**
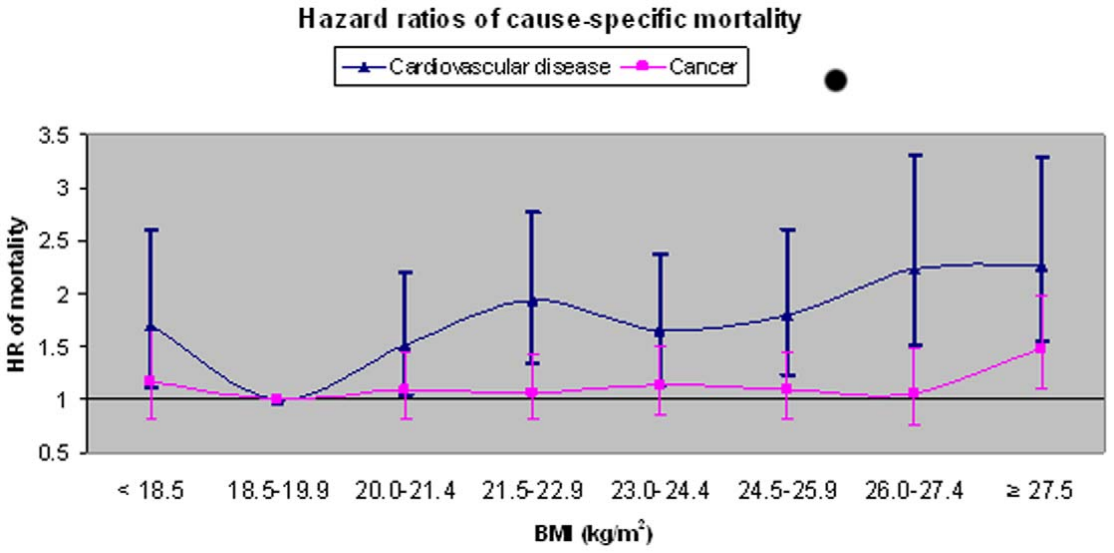
Hazard ratios of cause-specific mortality according to BMI. Hazard ratios of cause specific mortality in non-smokers without reported prevalent disease and excluding deaths occurring within 5 years of baseline (N = 30,538). Models adjusted for age, sex, year of enrollment, dialect, education, dietary pattern score and physical activity. Points represent hazard ratio (HR) point estimate and error bars represent 95% confidence intervals. P for linear association for cardiovascular disease  = 0.0001. P trend for linear association for cancer  = 0.04. Respective mortality counts for cancer and cardiovascular mortality by BMI (<18.5, 18.5–19.9, 20–21.4, 21.5–22.9, 23–24.4, 24.5–25.9, 26–27.4, ≥27.5): Cancer (58, 75, 130, 146, 146, 112, 64, 117), Cardiovascular disease (46, 39, 96, 139, 111, 99, 72, 95). Respective age & sex standardized mortality rates per 10,000 years follow up: Cancer (23, 18, 20, 20, 21, 21, 20, 29) and Cardiovascular disease (18, 9, 15, 19, 16, 19, 23, 23).

Cause-specific analyses examining total cardiovascular disease (CVD) mortality and cancer mortality are presented in **Figure 1**. We further examined components of CVD mortality in ischemic heart disease (IHD) (ICD-9 410–414, 427.5) and found risk significantly increased at BMI's>26.0 (p = 0.019 for linear association); and for cerebrovascular disease (CERE) mortality (ICD-9 430–438) risk increased at BMI's>27.5 (p = 0.04 for linear association) (data not presented). Comparatively, the absolute number of deaths, and the age and sex standardized mortality rates per 10,000 person years of follow up were significantly higher across the spectrum of BMI for ischemic heart disease vs. cerebrovascular disease (IHD rates- 10, 6, 7, 10, 8, 9 11,12 and CERE rates- 4, 3, 6, 6, 6, 5, 6, 8) for BMI groups (<18.5, 18.5–19.9, 20.0–21.4, 21.5–22.9, 23.0–24.4, 24.5–25.9, 26.0–27.4, ≥27.5). In consideration of excess weight-related cancer mortality, an increased risk was observed at BMI's≥27.5, HR = 1.50, 95% CI (1.02–2.20).

**Figure 2:**
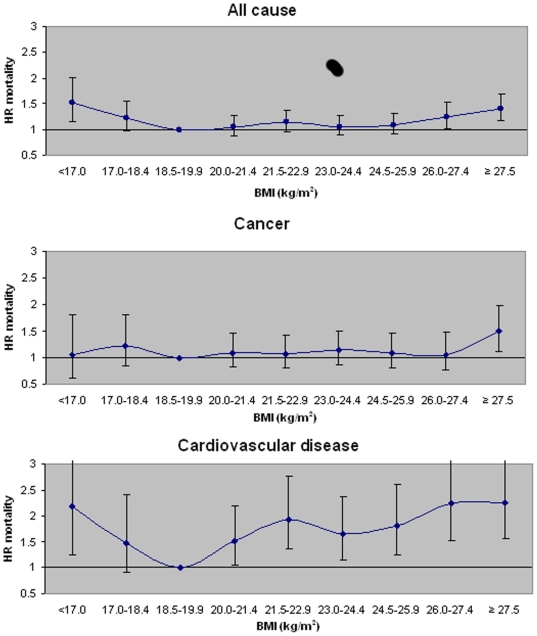
Hazard ratios of mortality incorporating BMI groups <18.5 kg/m^2^. Hazard ratios of all cause and cause specific mortality incorporating BMI groups less than 18.5 kg/m^2^ in non-smokers without reported prevalent disease and excluding deaths occurring within 5 years of baseline (N = 30,538); and adjusted for age, sex, year of enrollment, dialect, education, dietary pattern score and physical activity. Points represent hazard ratio (HR) point estimate and error bars represent 95% confidence intervals. P trend for quadratic association for all cause mortality = 0.0002. P trend for quadratic association for cardiovascular disease  = 0.0002. P trend for linear association for cancer  = 0.04.

**Figure 3:**
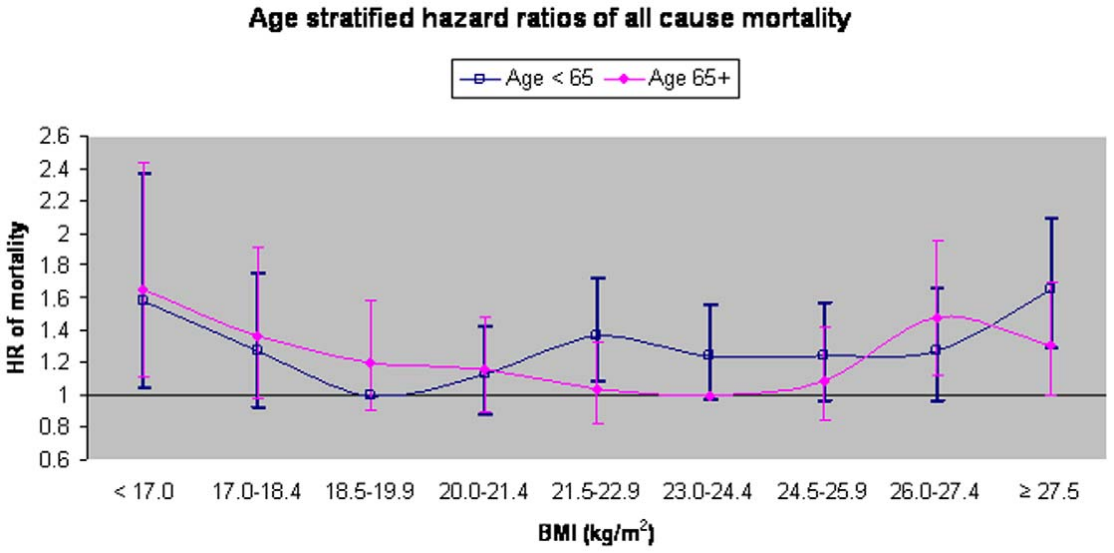
Age stratified hazard ratios of all-cause mortality according to BMI. Hazard ratios of all-cause mortality in participants aged less than 65 at baseline (N = 26,858, No. deaths = 1,344) and age 65+ (N = 3,680, No. deaths = 862). Figure encompasses all non-smokers who reported no prevalent disease and excludes deaths occurring within 5 years of baseline. Model is fully adjusted for age (continuous), sex, year of enrollment, dialect, education, dietary pattern score and physical activity. Points represent HR point estimate and error bars represent 95% confidence intervals. P value for interaction between age and BMI = 0.0001. Respective age & sex standardized mortality rates per 10,000 years follow up: Age<65 (48, 38, 28, 33, 40, 37, 39, 40, 51), Age 65+ (257, 219, 190, 194, 168, 161, 175, 226, 198).

All-cause and cause-specific data from examination of the low end of the BMI spectrum is presented in **Figure 2**. Data from an age-stratified analysis is presented in **Figure 3**. There is physiological rationale for age stratification as well as statistical evidence that the association differed for persons aged 65 and greater (P = 0.0001 for age × BMI interaction). The lowest age and sex-standardized all-cause mortality rate for ages 65 and greater is in BMI's 23–24.4, shifting the nadir of the BMI-mortality curve to the right relative to persons aged <65 at baseline. Cause-specific age stratified analyses show that the nadir of the curve did not shift in any of the cardiovascular related mortality groups (BMI = 18.5–19.9). However, for persons aged 65 or greater the lowest rate for cancer shifted to BMI's 23–24.4. Data on specific risks are not presented for age-stratified cause-specific analyses due to small numbers causing unstable estimates.

Excluding extreme BMI's (<15.5 and >35.0) did not alter the results in any specific analysis; nor was there any evidence the association differed by sex (P interaction = 0.36) across analyses. However, men had higher age standardized mortality rates per 10,000 person years of follow up in current, ex and non-smokers. In **Figure S1** data from a sex stratified analysis is presented and the shape and nature of the association largely mirrors the main results. A further examination of the shape of the association between BMI and mortality is presented in non-parametric graphs in **File S1**. These results provide visual evidence for the nature and shape of the association using different modeling assumptions.

## Discussion

In this large prospective study of men and women Chinese Singaporeans, aiming to inform on the optimal BMI range in relation to mortality for public health recommendations, we observed markedly greater mortality rates by BMI group in ex and current smokers compared to non-smokers. In non-smokers the lowest all-cause mortality rate was in BMI's 18.5–19.9 and risk of mortality significantly increased at BMI's<18.5 and ≥26.0. In ex-smokers the nadir of the BMI-mortality curve shifted right with the lowest all-cause mortality rate in BMI's 23.0–24.4 with similar points of increased risk as non-smokers. Among current smokers the nadir of the BMI-mortality curve shifted further right on the BMI spectrum with the lowest rate of all-cause mortality in BMI's 26.0–27.4 and associations of increased risk at BMI's<23.0 and ≥27.5. In cause-specific analyses examining total CVD mortality in non-smokers risk increases at BMI's≥20.0 and also at BMI's<18.5, and risk of cancer mortality increases at BMI's≥27.5. Sex stratified analyses show largely similar results between sexes, although men have higher mortality rates across analyses. Investigation of the low end of the BMI spectrum in non-smokers suggests that participants with BMI's<17.0 drive the increased risk in the WHO underweight category (<18.5). In analyses stratified by age, the lowest mortality rate and risk for adults less than 65 years of age is in BMI's 18.5–21.4 kg/m^2^; although the increases in risk for BMI values 21.5–27.5 are modest. In adults 65 years and older the lowest rate and risk of mortality is in BMI's 21.5–24.4 kg/m^2^. Independent of smoking and age, obesity status (BMI≥27.5) is clearly associated with increased risk for mortality, as is underweight status (BMI's<18.5).

The associations observed for CVD mortality are similar to what we and others have reported for BMI, diabetes and CVD risk, where values in the normal range of BMI associate with increasing risk. [10], [27] The risk of death from cancer and excess weight related cancers increased similarly in obese participants (BMI≥27.5 at enrollment). Overall, counts of cancer related mortality were greater and standardized mortality rates were slightly greater compared to cardiovascular disease. Substantial evidence supports the biologic plausibility of excess weight as a major risk factor in hastening chronic disease and thus risk for death. [10], [28]–[30] Indeed, the biological plausibility and negative effects of low BMI and health conditions and mortality have also been documented. [17].

Our results showing overweight ex-smokers (BMI = 23–24.4) and current smokers who were nearly obese (BMI = 26–27.4), as having the lowest rates of mortality relative to other ex and current smokers, is an under-studied topic. These results suggest over-weight status in person with a history of smoking may be protective relative to being under and normal weight or obese. However, due to the lack of clinical data and complete health status interpretation should be cautious. We have observed a similar situation in relation to smoking and lung cancer in this cohort where overweight and obesity was associated with lower rates and risk of lung cancers in smokers relative to normal and under weight. [31] Related, overweight and obese people with established cardiovascular disease, have been shown to have a better prognosis compared with non-overweight/non-obese patients. [32] Similarly, our results in participants aged 65 and greater suggest that the lowest risk for mortality is in the BMI range 23–24.4 kg/m^2^. These different associations between BMI and mortality in older participants and ever and current smokers is a topic in need of further investigation. It also suggests many different immunological, physiological and metabolic pathways play an important role positively or aversely depending on body weight. [32]–[34]

The question of BMI and mortality has received significant attention in Western populations, [3] but significantly less research has addressed the topic in Asian populations. The Shanghai Women's Health study found a monotonic association between baseline BMI and all-cause and cause-specific (CVD and cancer) mortality with the optimum level of the BMI range in non-smokers, without prevalent disease, and follow up greater than three years being <24.4, as risk increased significantly above this level compared to the referent of BMI's<22.2. [7] Gu et al. [8] examined the question in a nationally representative sample of Chinese men and women aged 40 years or older. Their data show the range of BMI not associated with increased risk of mortality being 24–26.9 in a healthy sub-sample of non-smokers, non-heavy drinkers and no prevalent disease.

In a Korean cohort of men and women aged 30–95 years, [9] a J-shaped association was observed for all cause mortality independent of smoking status in persons aged less than 64. For participants greater than 64 years of age at baseline there was no association between BMI and mortality. A J-shaped association was also observed in cancer deaths, but a monotonic linear association was observed in cardiovascular disease with the lowest risk at BMI's<18.5. Our results are comparable to this study in the age range less than 64 for all-cause mortality and cause specific trends; however, we did not have an extended upper age range at baseline, which may explain our different findings in this group. In a similar Korean cohort of women [6] a U-shaped association was observed with increased risk of mortality in BMI's<21 and BMI's≥27.0 compared to the referent group of BMI = 21–22 in the model adjusting for smoking status and accounting for deaths early in the follow up time. We observed similar results in non-smokers, but with a downward shift of an optimum BMI on the spectrum from 21–22 to 18.5–19.9 in Singaporean Chinese.

In a Japanese population of men and women aged 40–59 years, [5] risk increased in men with BMI's<23, but not higher BMI's after excluding the first 5 years of follow up and thorough adjustment including smoking status, compared to BMI's 23–24.9 (middle BMI category). In the comparative group of women BMI's<19 and >30 displayed an increased risk. A similar association was observed in never-smoking women. In non-smoking men the only increased risk was at BMI's<19, but this point estimate should be interpreted cautiously due to having a small number of cases. In a cohort study of Chinese men in Shanghai aged 45–64 lifetime never-smokers with BMI's<18.5 and >26.0 had an increased risk of mortality. In ever-smokers and current smokers there was no association. Our main results are comparable in non-smokers, however we found significant associations in both ever and current smokers.

In summary, the studies examining BMI and mortality in East and Southeast Asian populations present somewhat diverging optimum levels of BMI for lowest all-cause mortality. The nature of the association varies ranging from monotonic to J or U shaped, to nearly a reverse J-shape in Japanese men. [5] These inconsistencies are likely due to different methodological approaches, as well as different limitations across the studies. One methodological aspect that could help in comparability and interpretation of future studies of Asian populations is stratification on smoking status and clear presentation of age stratified data where BMI is a relevant surrogate measure of adiposity. Incorporating these methodological approaches may better inform on what an optimum level of BMI may be in relation to mortality in Asian adults. Objective choice of a referent group is another consideration that will help with comparability. Life course data on BMI and other measures in an Asian population would be ideal. Absent those data, consideration of the recent history as well as differences between and within the different populations of Asia in interpretation of the BMI-mortality association may still be informative.

In relation to public health and medical recommendations on relative weight, our data provide evidence that Chinese Southeast Asians distribute differently across the BMI spectrum vs. Western populations and thus categorizing their population level risk differs as the optimum relative weight is shifted to the left on the BMI spectrum. Applying Western based population cut-points categorizing overweight (>25.0 kg/m^2^) and obesity (>30.0 kg/m^2^) to Asian populations such as this Singapore population appears inappropriate. As more data on the subject of adiposity and health is gathered in all populations, it is becoming clearer that optimum relative weight (BMI) for health and longevity may function in a somewhat narrow range. [3] Yet, this remains controversial as BMI often behaves as a non-linear continuum of risk for chronic disease and mortality, and the nature of the association varies across populations and within populations as our study demonstrates. This risk needs to be considered with other health behaviors, socioeconomic, ethnic and geographic context in relation to health, as well as age considerations. Our data support a lower range of BMI cut-points in Southeast and East Asian populations relative to Western populations; however these differ by smoking and age status making it difficult to provide scientific rationale for a blanket population recommendation for BMI cut-points.

Limitations of our study include incomplete data on mental health conditions, other chronic health conditions such as COPD, cirrhosis and some neurodegenerative diseases at baseline. Further considerations include the use of self-reported height, weight and other demographic and lifestyle data. Nonetheless, BMI by self report has been shown to be highly valid in a number of populations. [10] Yet, misclassification and measurement error of height and weight need to be considered as possible influences on the results. Additional measures of body habitus may complement BMI in this population and contribute further to our understanding; as well as multiple assessments of relative weight over time in the etiology and epidemiology of body composition and mortality.

Strengths of our study include thorough assessment of potential lifestyle and demographic confounders of the BMI-mortality association, a large sample size and ample amount of events combined with a long follow up time. These study characteristics allowed us to stratify on important confounders and mediators and address these areas more thoroughly than previous studies on similar Asian populations. The participants are also representative of the source population and mortality assessment is virtually complete.

In conclusion, independent of age or BMI, smoking greatly increases the rate of mortality and influences the association between BMI and mortality. In non-smokers below the age of 65 the optimum level of BMI in relation to the lowest rate and risk of all-cause mortality appears to be between 18.5 and 21.4 kg/m^2^. For non-smokers over age 65 the corresponding optimal range of BMI with respect to all-cause mortality appears to be between 21.5 and 24.4 kg/m^2^. Our findings contribute to the science behind public health considerations on the appropriate and optimal range of relative weight in some Asian populations where a higher proportion of BMI values fall in a lower range compared to Western populations.

## Supporting Information

Table S1 (0.10 MB DOC)

Figure S1 (0.03 MB TIF)

File S1 An alternative approach to analyzing the data that examines the shape of the data w/o calculating risk.
(0.14 MB DOC)
