# Supplementary material for: BMI, All-Cause and Cause-Specific Mortality in Chinese Singaporean Men and Women: The Singapore Chinese Health Study
Source: PLoS One. 2010 Nov 15;5(11):e14000. doi: 10.1371/journal.pone.0014000 (PMC2981556; doi:10.1371/journal.pone.0014000)
Supplement: Table S1 — (0.10 MB DOC) [file pone.0014000.s001.doc]

| **Table S1: Standardized all-cause mortality rate and hazard ratios according to body mass index (BMI) in SCHS** | | | | | | | | | |
| --- | --- | --- | --- | --- | --- | --- | --- | --- | --- |
|  | Body Mass Index (kg/m^2^) | | | | | | | |  |
|  | **<18.5** | **18.5-19.9** | **20.0-21.4** | **21.5-22.9** | **23.0-24.4** | **24.5-25.9** | **26.0-27.4** | **≥27.5** |  |
| Current smokers including reported prevalent disease N=9,777, No. deaths=2,762 | | | | | | | | | |
| No. deaths/N | 402/1,191 | 419/1,348 | 484/1,738 | 447/1,639 | 385/1,480 | 287/1,036 | 141/584 | 197/761 | P trend |
| *Standardized rate | 296 | 269 | 234 | 231 | 216 | 232 | 201 | 215 |  |
| HR (95% CI) | 1.18 (0.97-1.43) | 1.14 (0.94-1.38) | 1.03 (0.86-1.25) | 0.99 (0.82-1.20) | 1.00 (0.83-1.21) | 1.09 (0.89-1.33) | 1.0 | 1.25 (1.01-1.55) | 0.0004 |
| Ex-smokers including reported prevalent disease N=5,708, No. deaths 1,423 | | | | | | | | | |
| No. deaths/N | 148/399 | 147/512 | 216/852 | 220/946 | 210/1,033 | 190/798 | 127/516 | 165/652 |  |
| *Standardized rate | 344 | 251 | 218 | 198 | 172 | 203 | 210 | 218 |  |
| HR (95% CI) | 1.48 (1.20-1.82) | 1.25 (1.02-1.55) | 1.12 (0.93-1.36) | 1.04 (0.86-1.26) | 1.0 | 1.17 (0.96-1.43) | 1.27 (1.02-1.59) | 1.36 (1.11-1.68) | <0.0001 |
| Non-smokers including reported prevalent disease N=35,766, No. deaths 4,171 | | | | | | | | | |
| No. deaths/N | 304/2,260 | 375/3,612 | 576/5,643 | 730/6,422 | 686/6,210 | 556/4,779 | 381/2,937 | 563/3,903 |  |
| *Standardized rate | 108 | 82 | 80 | 89 | 87 | 91 | 103 | 115 |  |
| HR (95% CI) | 1.19 (1.03-1.39) | 1.0 | 0.93 (0.82-1.06) | 1.02 (0.90-1.15) | 1.00 (0.88-1.13) | 1.02 (0.89-1.16) | 1.16 (1.00-1.34) | 1.27 (1.12-1.45) | < 0.0001 |
| ^1^Current smokers excluding reported prevalent disease N=8,413, No. deaths=2,128 | | | | | | | | | |
| No. deaths/N | 333/1,064 | 337/1,203 | 371/1,525 | 356/1,416 | 291/1,255 | 206/860 | 88/465 | 146/625 |  |
| *Standardized rate | 270 | 238 | 200 | 209 | 189 | 197 | 153 | 191 |  |
| HR (95% CI) | 1.40 (1.11-1.77) | 1.32 (1.05-1.67) | 1.17 (0.93-1.47) | 1.19 (0.94-1.51) | 1.13 (0.89-1.43) | 1.23 (0.96-1.58) | 1.0 | 1.44 (1.10-1.87) | 0.001 |
| ^1^Ex-smokers excluding reported prevalent disease N=4,333, No. deaths=833 | | | | | | | | | |
| No. deaths/N | 99/313 | 88/402 | 130/654 | 126/732 | 114/777 | 102/598 | 75/387 | 96/470 |  |
| *Standardized rate | 284 | 184 | 165 | 142 | 120 | 140 | 161 | 171 |  |
| HR (95% CI) | 1.69 (1.29-2.21) | 1.26 (0.96-1.67) | 1.23 (0.95-1.58) | 1.10 (085-1.42) | 1.0 | 1.16 (0.89-1.52) | 1.48 (1.10-1.98) | 1.51 (1.15-1.98) | <0.0001 |
| ^1^Non-smokers excluding reported prevalent disease N=31,176, No. deaths=2,844 | | | | | | | | | |
| No. deaths/N | 225/2,024 | 262/3,271 | 417/5,030 | 505/5,681 | 453/5,359 | 377/4,117 | 255/2,496 | 350/3,198 |  |
| *Standardized rate | 87 | 62 | 64 | 69 | 66 | 71 | 80 | 86 |  |
| HR (95% CI) | 1.27 (1.06-1.52) | 1.0 | 0.99 (0.85-1.15) | 1.06 (0.91-1.23) | 1.01 (0.87-1.18) | 1.04 (0.89-1.22) | 1.19 (1.01-1.42) | 1.25 (1.07-1.47) | 0.0002 |

Continued

|  | **<18.5** | **18.5-19.9** | **20.0-21.4** | **21.5-22.9** | **23.0-24.4** | **24.5-25.9** | **26.0-27.4** | **≥27.5** |  |
| --- | --- | --- | --- | --- | --- | --- | --- | --- | --- |
| ^2^Current smokers excluding deaths with < 5 years follow up time N=7,853, No. deaths=1,568 | | | | | | | | | |
| No. deaths/N | 232/963 | 248/1,114 | 279/1,433 | 269/1,329 | 219/1,183 | 151/805 | 59/436 | 111/590 |  |
| *Standardized rate | 192 | 178 | 153 | 160 | 144 | 147 | 104 | 147 |  |
| HR (95% CI) | 1.52 (1.14-2.02) | 1.50 (1.13-1.99) | 1.34 (1.01-1.77) | 1.38 (1.04-1.83) | 1.27 (0.95-1.69) | 1.36 (1.00-1.83) | 1.0 | 1.65 (1.20-2.26) | 0.015 |
| ^2^Ex-smokers excluding deaths with < 5 years follow up time N=4,108, No. deaths=605 | | | | | | | | | |
| No. deaths/N | 68/282 | 61/375 | 88/612 | 98/704 | 86/749 | 77/573 | 54/366 | 73/447 |  |
| *Standardized rate | 200 | 130 | 114 | 111 | 91 | 107 | 117 | 132 |  |
| HR (95% CI) | 1.66 (1.20-2.28) | 1.21 (0.87-1.68) | 1.13 (0.84-1.52) | 1.15 (0.86-1.54) | 1.0 | 1.16 (0.85-1.57) | 1.43 (1.01-2.01) | 1.56 (1.14-2.13) | 0.0003 |
| ^2^Non-smokers excluding deaths with < 5 years follow up time N=30,538, No. deaths 2,206 | | | | | | | | | |
| No. deaths/N | 170/1,969 | 191/3,200 | 326/4,939 | 400/5,576 | 350/5,256 | 291/4,031 | 195/2,436 | 283/3,131 |  |
| *Standardized rate | 66 | 46 | 51 | 55 | 51 | 55 | 61 | 70 |  |
| HR (95% CI) | 1.32 (1.07-1.62) | 1.0 | 1.06 (0.88-1.26) | 1.15 (0.96-1.36) | 1.07 (0.89-1.27) | 1.09 (0.91-1.31) | 1.25 (1.02-1.53) | 1.40 (1.16-1.68) | 0.0009 |

SCHS= Singapore Chinese Health Study

*Standardized rate=Age and sex standardized mortality rate per 10,000 person years using person year time, age & sex distributions of SCHS

HR (95% CI) = Hazard Ratio; 95 % confidence interval: Model adjusted for age, sex, year of enrollment, dialect, education, dietary pattern score, and physical activity (and intensity and duration of smoking in smokers plus duration since quit in ex-smokers)

*P* trend= *P* value for quadratic term (U or J shaped) using median of BMI in each category as continuous variable

^1^Exclusion of 4,590 participants with prevalent cardiovascular disease, diabetes mellitus, and respiratory disease

^2^Exclusion of prevalent disease + participants who died within 5 years o
