# Supplementary figures and images for: BMI, All-Cause and Cause-Specific Mortality in Chinese Singaporean Men and Women: The Singapore Chinese Health Study
Source: PLoS One. 2010 Nov 15;5(11):e14000. doi: 10.1371/journal.pone.0014000 (PMC2981556; doi:10.1371/journal.pone.0014000)

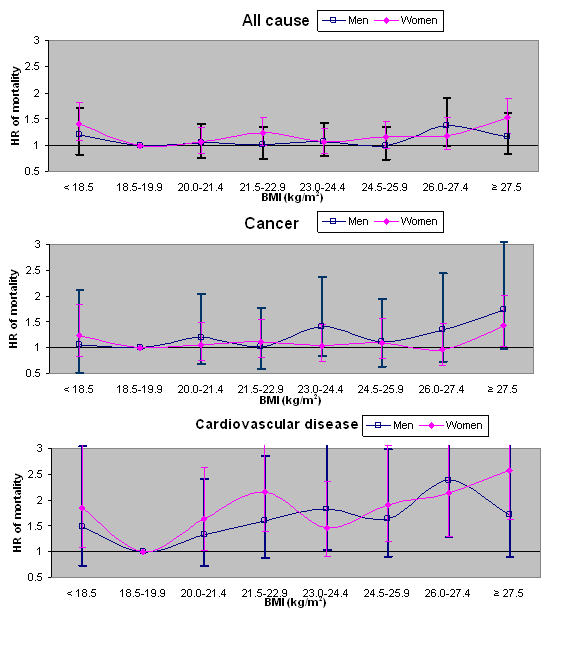

Supplement: Figure S1 — (0.03 MB TIF) [file pone.0014000.s002.tif]
