# Supplementary material for: BMI, All-Cause and Cause-Specific Mortality in Chinese Singaporean Men and Women: The Singapore Chinese Health Study
Source: PLoS One. 2010 Nov 15;5(11):e14000. doi: 10.1371/journal.pone.0014000 (PMC2981556; doi:10.1371/journal.pone.0014000)
Supplement: File S1 — An alternative approach to analyzing the data that examines the shape of the data w/o calculating risk. (0.14 MB DOC) [file pone.0014000.s003.doc]

**Supporting documents: Non-parametric methods and figures**

We further examined the relationship between BMI, all-cause and cause-specific mortality by looking at the shape of the association with a non-parametric (local polynomial) regression analysis using the statistical package ‘R’ version 2.4.1. The purpose of such analysis is to produce a trend graph taking advantage of the large sample size and as an exploratory procedure to confirm the initial analysis using Cox regression methods.^1^ The log odds of mortality and cause specific mortality are modeled with a generalized additive model (GAM) specifying BMI as a loess fit,^2^ and adjusting for the same possible confounders as in the above Cox regression model including adjustment for person-years time. In contrast to the Cox models, there is no reference group, as the parameters are estimated using local fitting while controlling for the degree of smoothing and polynomial fit.^2^ The degree of smoothing was set at a modest parameter (0.5 of 1.0, where 1.0 is the highest level of smoothing) and the polynomial degree was set to a curvilinear fit for each model.

The graphs below represent the non-smoking population who reported no baseline prevalence of the chronic diseases/conditions surveyed, with exclusion of deaths within five years of baseline (N=30,538). We also present two graphs of ex and current smokers who reported no baseline prevalence of the chronic diseases/conditions surveyed, with exclusion of deaths within five years of baseline. The solid line represents the point estimate of the curve, and the dotted lines represent the upper and lower 95% confidence bands. The shaded area at the bottom of the plot is the distribution of BMI data.

**References:**

**1.** Rothman KJ, Greenland S, Lash TL. *Modern Epidemiology*. Philadelphia: Lippincott Williams & Wilkins; 2008.

**2.** Hastie T, Tibshirani R. *Generalized Additive Models* London: Chapman and Hall; 1990.
